# Klotho-mediated activation of the anti-oxidant Nrf2/ARE signal pathway affects cell apoptosis, senescence and mobility in hypoxic human trophoblasts: involvement of Klotho in the pathogenesis of preeclampsia

**DOI:** 10.1186/s13008-024-00120-2

**Published:** 2024-04-17

**Authors:** Baomei Xu, Fang Cheng, Xiaolei Xue

**Affiliations:** Obstetrical Department, The Fifth Affiliated Hospital of Xinjiang Medical University, Henan Road No. 118, Urumqi, 830000 Xinjiang China

**Keywords:** Preeclampsia, Oxidative stress, Human trophoblasts, Hypoxia, Nrf2/ARE signal pathway

## Abstract

The anti-aging gene Klotho is implicated in the pathogenesis of preeclampsia (PE), which is a pregnancy disease characterized by hypertension and proteinuria. Oxidative stress is closely associated with the worse outcomes in PE, and Klotho can eliminate Reactive Oxygen Species (ROS), but it is still unclear whether Klotho regulates PE pathogenesis through modulating oxidative damages. Here, by analyzing the clinical data, we found that Klotho was aberrantly downregulated in PE umbilical cord serum and placental tissues, compared to their normal counterparts. In in vitro experiments, the human trophoblasts were subjected to hypoxic pressure to establish the PE models, and we confirmed that hypoxia also decreased the expression levels of Klotho in those trophoblasts. In addition, through performing functional experiments, we confirmed that hypoxia promoted oxidative damages, cell apoptosis and senescence, whereas suppressed cell invasion in human trophoblasts, which were all reversed overexpressing Klotho. The following mechanical experiments verified that Klotho increased the levels of nuclear Nrf2, total Nrf2, SOD2 and NQO1 to activate the anti-oxidant Nrf2/ARE signal pathway, and silencing of Nrf2 abrogated the protective effects of Klotho overexpression on hypoxic human trophoblasts. Consistently, in in vivo experiments, Klotho overexpression restrained oxidative damages and facilitated cell mitosis in PE rats’ placental tissues. In conclusion, this study validated that Klotho activated the Nrf2/ARE signal pathway to eliminate hypoxia-induced oxidative damages, cell apoptosis and senescence to recover normal cellular functions in human trophoblasts, and our data supported that Klotho could be used as novel biomarker for PE diagnosis and treatment.

## Introduction

As one of the serious pregnancy diseases, preeclampsia (PE) often happens in the pregnancy women over 20th gestational weeks, and its clinical symptoms include hypertension and proteinuria [1, 2]. According to the newest data, PE affects about 7–10% of pregnant women worldwide and the mortality of this disease among pregnancies is very high [1, 2]. The pathogenesis of PE is very complicated, and researchers agree that dysfunctions of human trophoblasts play vital role in accelerating the development of this disease [3–5]. Mechanistically, malfunctions of trophoblasts will hinder the invasion and migration of this cell toward uterine spiral arteries, which will cause disordered neovascularization and abnormal placental development with hypoxia and ischemia, resulting in the aggravation of PE [3–5]. In addition, as previously described, hypoxic environment will initiate and enhance oxidative stress-related damages and cellular inflammation, which are considered as two important pathogenetic factors that aggravate the progression of PE [6]. Consistently, it is verified that the Nrf2/Keap1 pathway-mediated anti-oxidant effects play critical role in modulating PE pathogenesis [6]. However, the detailed molecular mechanisms are still largely unknown. In recent studies, it is found that hypoxia-induced oxidative damages in human trophoblasts is closely associated with the development of PE [7, 8]. For example, Liao et al. evidence that blockage of oxidative stress is effective to ameliorate pathogenesis of PE by suppressing ferroptosis [8], and Liu et al. report that elimination of Reactive Oxygen Species (ROS) by Chinese medicine Atractylenolide (ATL) recover cellular functions of human trophoblasts to attenuate PE development [7]. Therefore, it is important to search for the strategies that can eliminate ROS generation during PE initiation and progression.

Oxidative stress is verified as inducers for cell apoptosis [9, 10] and senescence [11, 12], which are considered as two important biological functions that contribute to the development of PE [13]. For example, Hu et al. report that cyclosporin A alleviates PE progression through suppressing trophoblast apoptosis and senescence [13]. In addition to above two biological functions, excessive oxidative damages also suppress invasion abilities of trophoblasts [14, 15]. As previously described, the development of PE is influenced by a large cohort of genes [16–18], and the anti-aging gene Klotho is reported to be closely associated with the development of various diseases, including PE [19, 20]. For instance, Wang et al. investigate the correlations of Klotho polymorphisms with PE progression [20], Cecati rt al. indicate the potential role of placental Klotho in the pathogenesis of PE [19], and Uzun et al. suggest that placental and serum Klotho can be used as sensitive biomarker for server PE [21]. However, all the published literatures are correlation analysis, and the detailed functions and mechanisms by which Klotho regulates the development of PE have not been studied. In addition, Klotho is implicated in the regulation of oxidative stress [22, 23], cell apoptosis [22, 24], senescence [25, 26] and migration [27, 28], suggesting that Klotho may play critical role in regulating PE development.

It is widely known that activation of the anti-oxidant Nrf2/ARE signal pathway plays central role in sustaining the homeostasis of oxidative stress [29–31]. Mechanistically, the Nrf2 combines with Keap1 to form the Keap1-Nrf2 complex in cytoplasm under normal conditions. Once the oxidative stress occurs and ROS accumulation initiates, the Keap1-Nrf2 complex will be disintegrated and Nrf2 is dispatched to nucleus to exert its anti-oxidant effects and eliminate ROS [29–31]. Of note, the Nrf2/Keap1 signal pathway is characterized with multifaceted functions which participates in the regulation of multiple diseases, especially in cancer, and previous publications suggest that Nrf2/Keap1 plays critical role in regulating the initiation, progression and aggravation of prostate cancer [32], ovarian cancer [33] and cervical endometrial cancers [34]. Interestingly, it is reported that activation of the Nrf2/ARE signal pathway ameliorates PE progression [8, 35], and Nrf2 itself can regulate cell apoptosis [36], senescence [37] and migration [38], indicating that Nrf2 is critical for PE pathogenesis. Surprisingly, recent data suggest that Klotho is involved in the regulation of Nrf2/ARE signal pathway [25, 39]. For instance, Chen et al. verify that Klotho-deficiency causes heart aging via modulating the Nrf2/ARE signal pathway [25], and Xing et al. evidence that Klotho activates the Nrf2 signal pathway in podocytes to ameliorate diabetic nephropathy [39]. Therefore, it is reasonable to speculate that Klotho may affect PE pathogenesis through modulating Nrf2/ARE signal pathway-mediated anti-oxidant effects, but the detailed mechanisms are still largely unknown.

Collectively, based on the published data, the present study aims to investigate the functions of Klotho in regulating the development PE, and uncover the potential underlying mechanisms. Our findings suggested that Klotho was a novel indicator for PE diagnosis and therapy, and overexpression of Klotho re-activated the anti-oxidant Nrf2/ARE signal pathway to restrain oxidative stress, apoptosis and cellular senescence, and facilitated cell invasion in hypoxic human trophoblasts, and the Klotho/Nrf2/ARE signal pathway was firstly identified as critical regulators for PE pathogenesis.

## Results

### The expression status of Klotho was closely associated with preeclampsia development

**Fig. 1:**
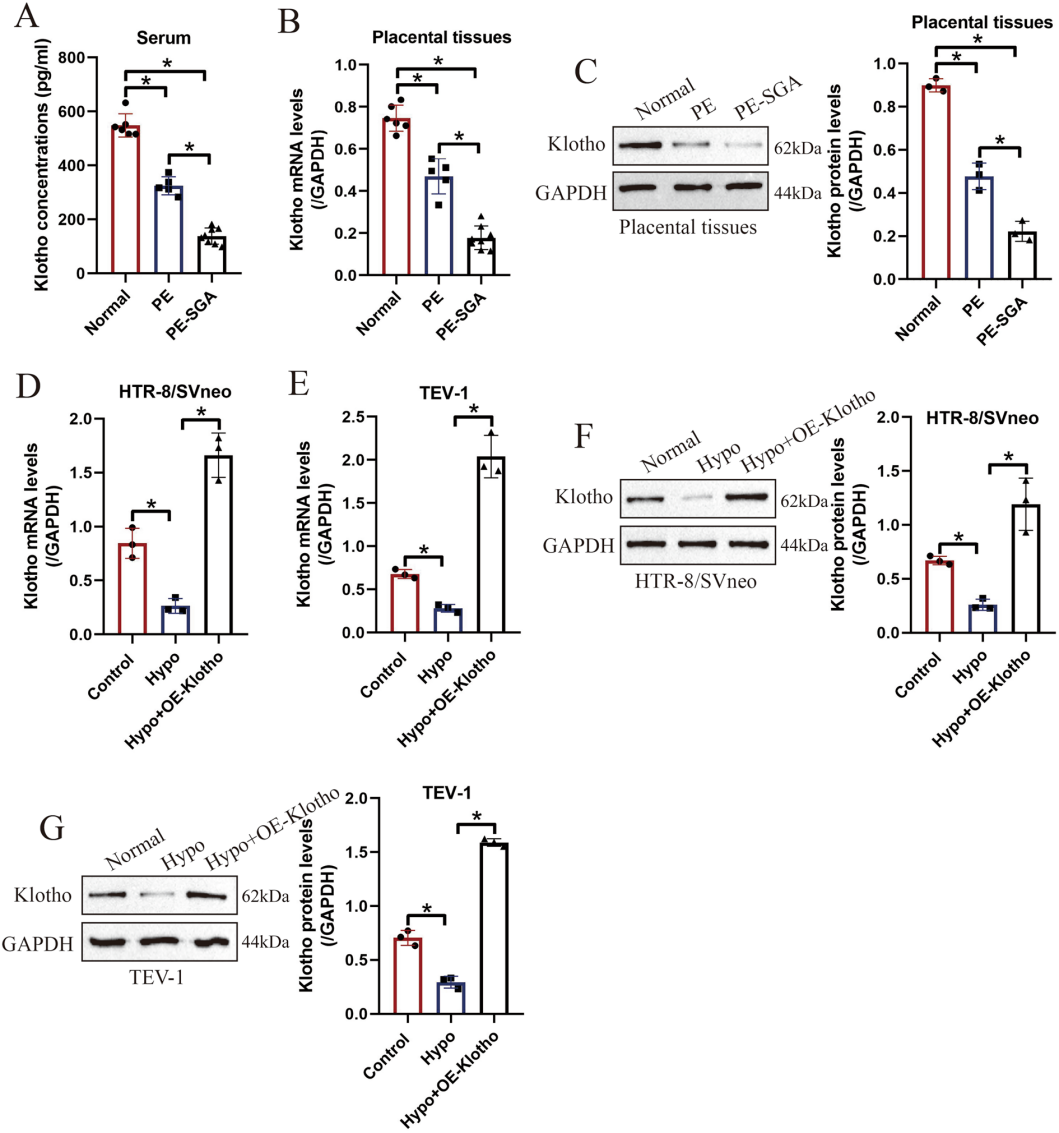
Klotho was correlated with the development of PE in clinic and in vitro. **A** The contents of Klotho in PE patients’ serum were determined by ELISA analysis. The mRNA and protein levels of Klotho in PE patients’ placental tissues were respectively determined by performing **B** Real-Time qPCR and **C** Western Blot analysis. The human trophoblasts were subjected to hypoxia treatment, and **D**, **E** Real-Time qPCR and **F**, **G** Western Blot was used to detect the expression levels of Klotho protein in those cells in vitro. Each experiment was repeated at least for three times, and *P < 0.05 was considered as statistical significance. Each experiment was repeated at least for three times

To determine the correlations of Klotho levels with the development of preeclampsia (PE), we initially collected the maternal and umbilical cord serum from normal participants, PE pregnancy with or without small for gestational age infant (PE-SGA). The ELISA analysis results showed that Klotho was downregulated in the PE patients’ serum compared to the normal volunteers, and Klotho was especially low-expressed in the PE-SGA patients’ serum (Fig. 1A). The above results were confirmed by the following Real-Time qPCR (Fig. 1B) and Western Blot analysis (Fig. 1C) that Klotho tended to be low-expressed in the PE and PE-SGA patients’ placental tissues, which were in consistent with the ELISA analysis results, suggesting that Klotho was associated with the development of PE in clinic. In addition, according to the protocols provided by the previous literatures, the human trophoblasts (HTR-8/SVneo and TEV-1) were subjected to hypoxia to establish the PE models in vitro, and our data suggested that hypoxia decreased the mRNA levels of Klotho in human trophoblasts (Fig. 1D, E), which were supported by the following Western Blot analysis that hypoxia also suppressed Klotho protein expressions in human trophoblasts (Fig. 1F, G). The above clinical and pre-clinical data suggested that Klotho was related with the initiation and development of PE.

### Overexpression of Klotho ameliorated hypoxia-induced detrimental effects in human trophoblasts

**Fig. 2:**
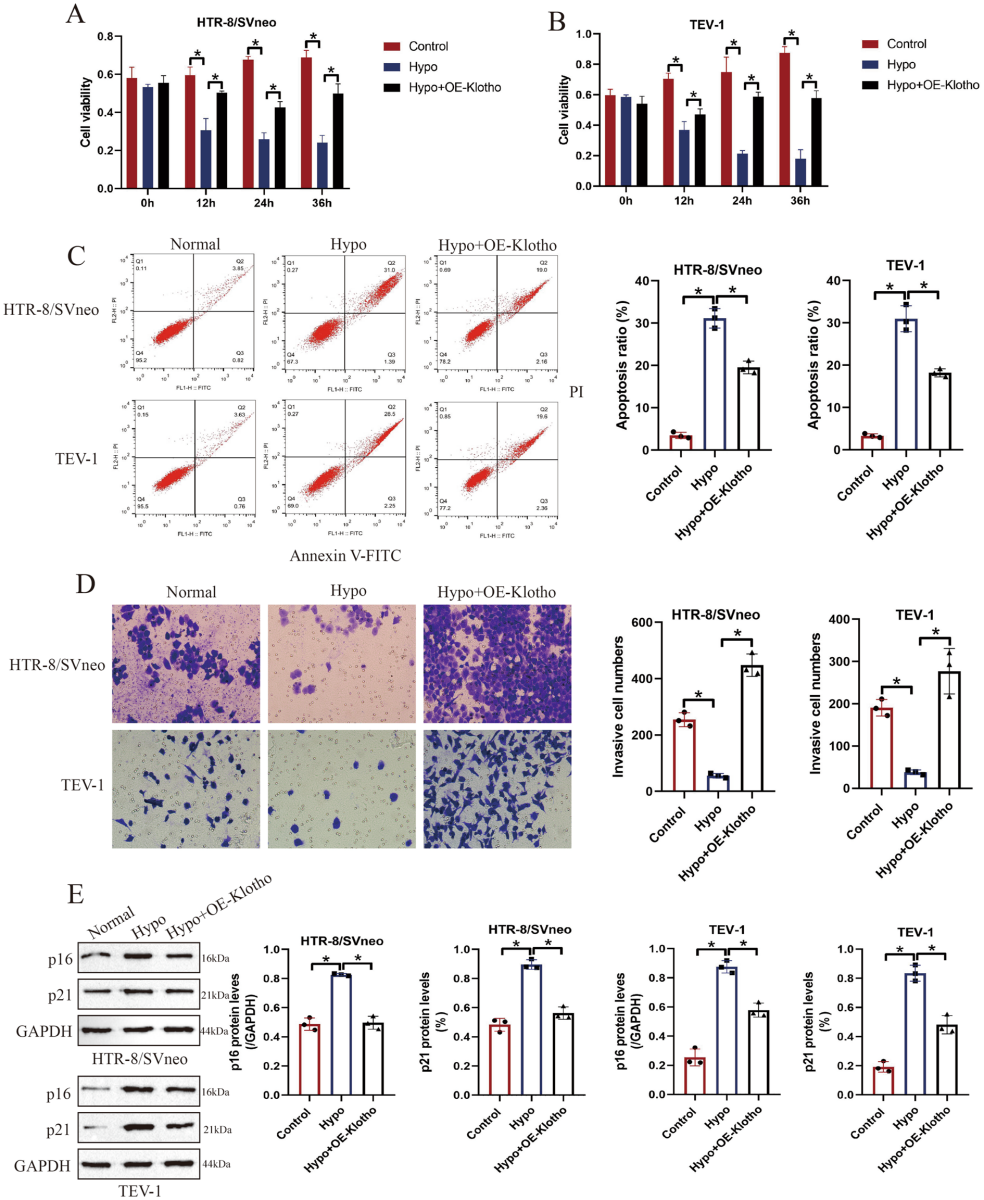
Overexpression of Klotho reversed the regulating effects of hypoxia on cell viability, apoptosis, invasion and senescence. **A**, **B** The human trophoblasts were subjected to hypoxia, and cell viability was determined by performing MTT assay. **C** The Annexin V-FITC/PI double staining method was employed to detect cell apoptosis of human trophoblasts. **D** Cell invasion abilities of HTR-8/SVneo and TEV cells were determined by performing Transwell assay. **E** Western Blot analysis was used to analyze the expression status of the cellular senescence-related proteins (p16 and p21) in human trophoblasts. Each experiment was repeated at least for three times, and *P < 0.05 was considered as statistical significance. Each experiment was repeated at least for three times

Considering that Klotho was aberrantly downregulated in PE models, to verify its biological functions, the Klotho overexpression vectors were delivered into the HTR-8/SVneo and TEV-1 cells, and the transfection efficiency was determined by performing Real-Time qPCR (Fig. 1D, E) and Western Blot analysis (Fig. 1F, G). Then, the human trophoblasts were subjected to hypoxic conditions, and the MTT assay results showed that hypoxia suppressed cell viability in a time-dependent manner, which were reversed by overexpressing Klotho (Fig. 2A, B). The above results were supported by the FCM assay that hypoxia-induced apoptotic cell death in human trophoblasts were also reversed by upregulating Klotho (Fig. 2C). Then, we performed Transwell assay to determine cell mobility, and the results suggested that overexpression of Klotho facilitate cell invasion in hypoxic human trophoblasts (Fig. 2D). In addition, we verified that hypoxia also upregulated p16 and p21 to trigger cellular senescence in human trophoblast, which were suppressed by upregulating Klotho (Fig. 2E). The above data suggested that Klotho overexpression was capable of ameliorating hypoxia-induced detrimental effects on human trophoblasts through modulating cell apoptosis, senescence and invasion.

### Klotho activated the Nrf2/ARE signal pathway to eliminate hypoxia-induced oxidative damages in human trophoblasts

**Fig. 3:**
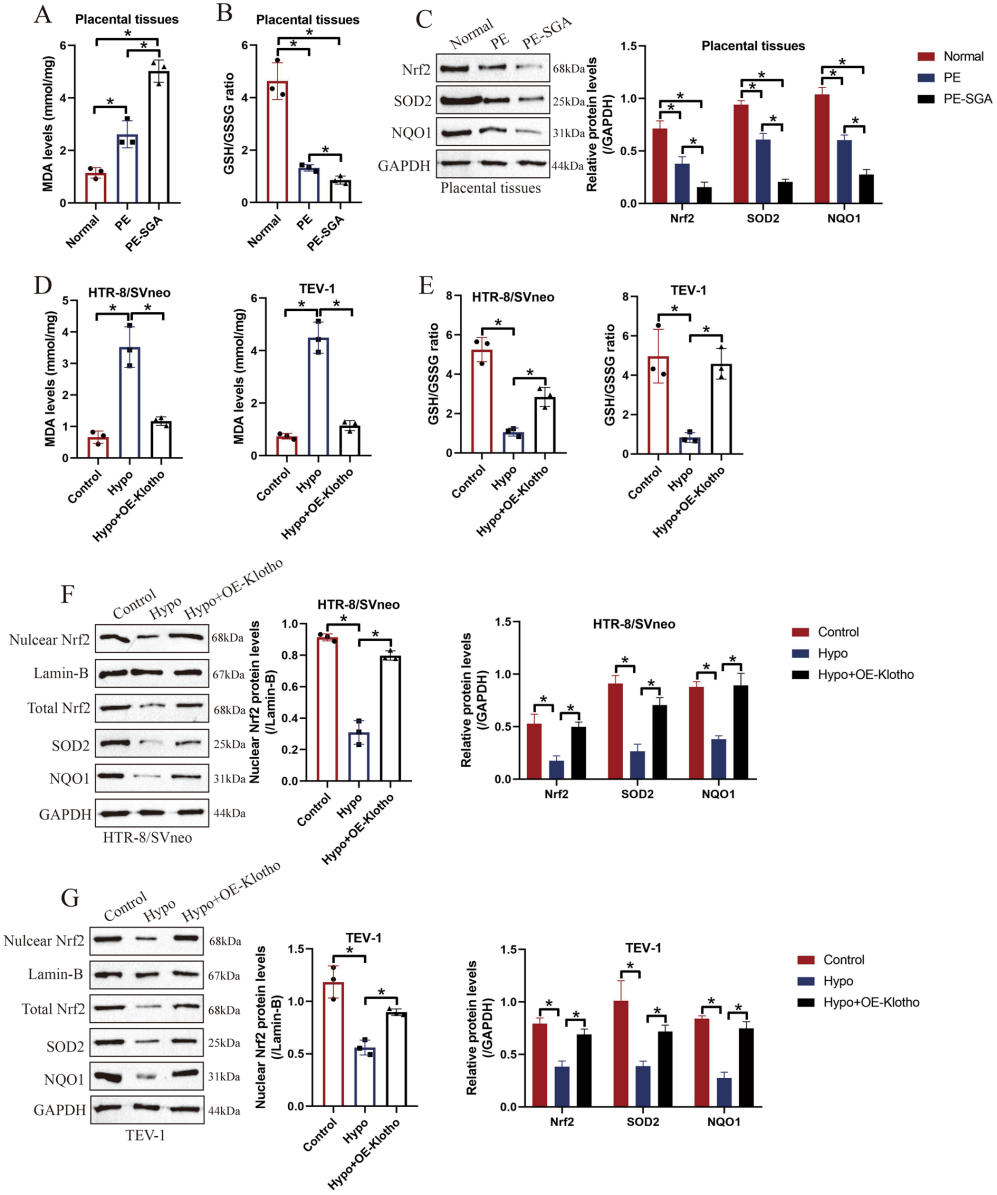
Klotho regulated oxidative stress in PE models. **A** The MDA levels and **B** GSH/GSSG ratio in PE patients’ placental tissues were determined by their corresponding detection kits. **C** The expression levels of anti-oxidant proteins (Nrf2, SOD2 and NQO1) in PE patients’ placental tissues were detected by performing Western Blot analysis. **D** The MDA levels and **E** GSH/GSSG ratio in hypoxic human trophoblasts were respectively determined by their kits. **F**, **G** The human trophoblasts were subjected to hypoxia, and Western Blot analysis was performed to examine the expression status of nuclear Nrf2, total Nrf2, SOD2 and NQO1 in the cells. Each experiment was repeated at least for three times, and *P < 0.05 was considered as statistical significance. Each experiment was repeated at least for three times

Oxidative stress-induced cell damages are considered as critical factors that contribute to the aggravation of PE [7, 8], which were supported by our data that MDA levels were upregulated (Fig. 3A), whereas GSH/GSSG ratio (Fig. 3B), Nrf2, SOD2 and NQO1 (Fig. 3C) were downregulated in PE and PE-SGA patients’ placental tissues, compared to the tissues collected from normal volunteers. The above results were in consistent with the in vitro experiments, which showed that hypoxia also increased MDA levels (Fig. 3D), but suppressed GSH/GSSG ratio (Fig. 3E), nucleic and cytoplastic Nrf2, SOD2 and NQO1 (Fig. 3F, G) to trigger oxidative damages in both HTR-8/SVneo and TEV-1 cells, and hypoxia-induced oxidative stress could be restrained by upregulating Klotho (Fig. 3D–G). The above data hinted that oxidative stress was closely associated with PE development, and Klotho activated the anti-oxidant Nrf2/ARE signal pathway to restrain hypoxia-induced oxidative damages in human trophoblasts.

### Klotho Nrf2/ARE signal pathway-dependently regulated cell apoptosis, senescence and mobility in hypoxic human trophoblasts

**Fig. 4:**
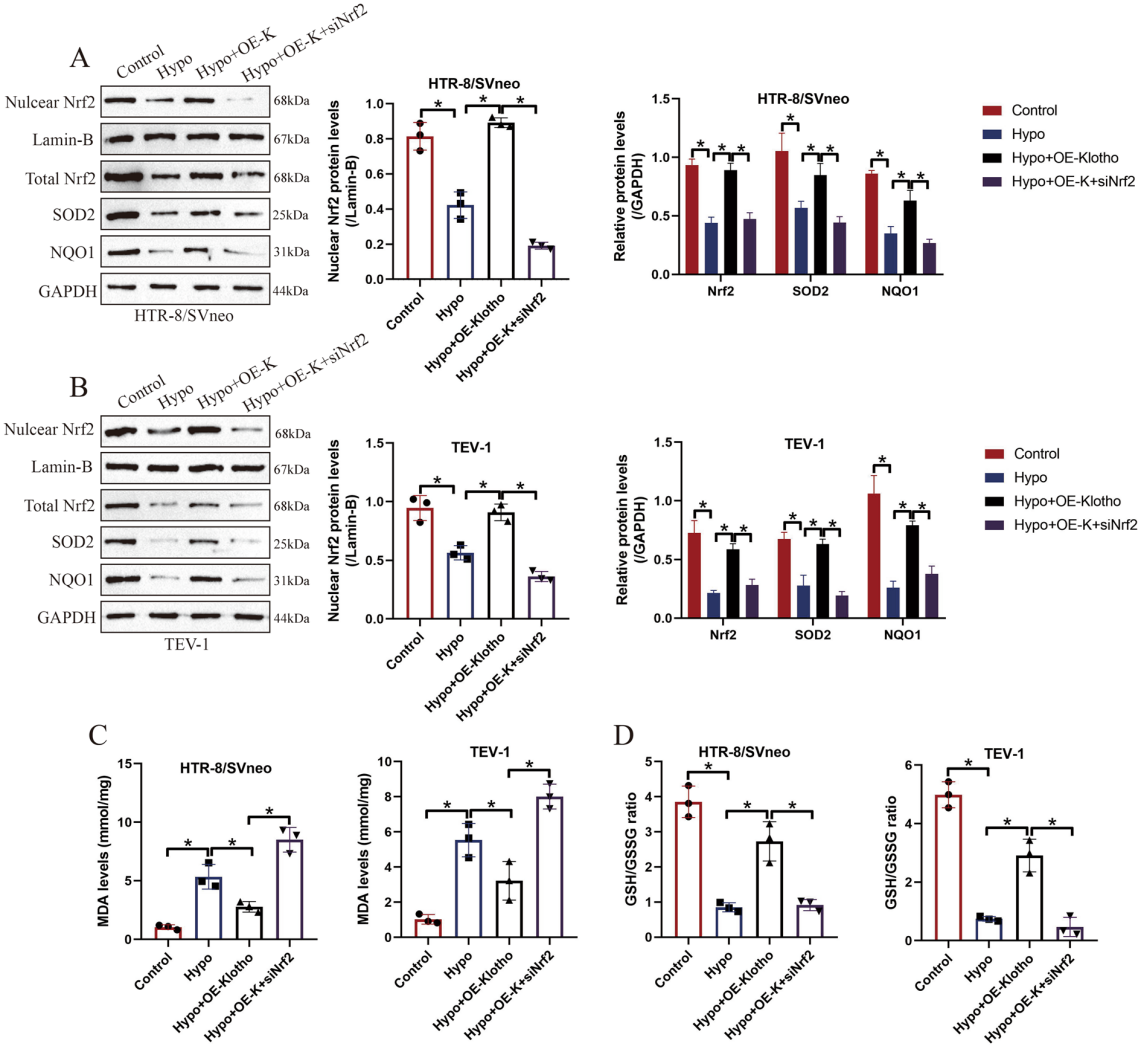
Silencing of Nrf2 re-triggered oxidative damages in Klotho-overexpressed hypoxic human trophoblasts. **A**, **B** The regulating effects of Nrf2 knockdown on the expression levels of nuclear Nrf2, total Nrf2, SOD2 and NQO1 were determined by performing Western Blot analysis. **C** The MDA levels and **D** GSH/GSSG ratio in human trophoblasts were determined by using their corresponding kits. Each experiment was repeated at least for three times, and *P < 0.05 was considered as statistical significance. Each experiment was repeated at least for three times

**Fig. 5:**
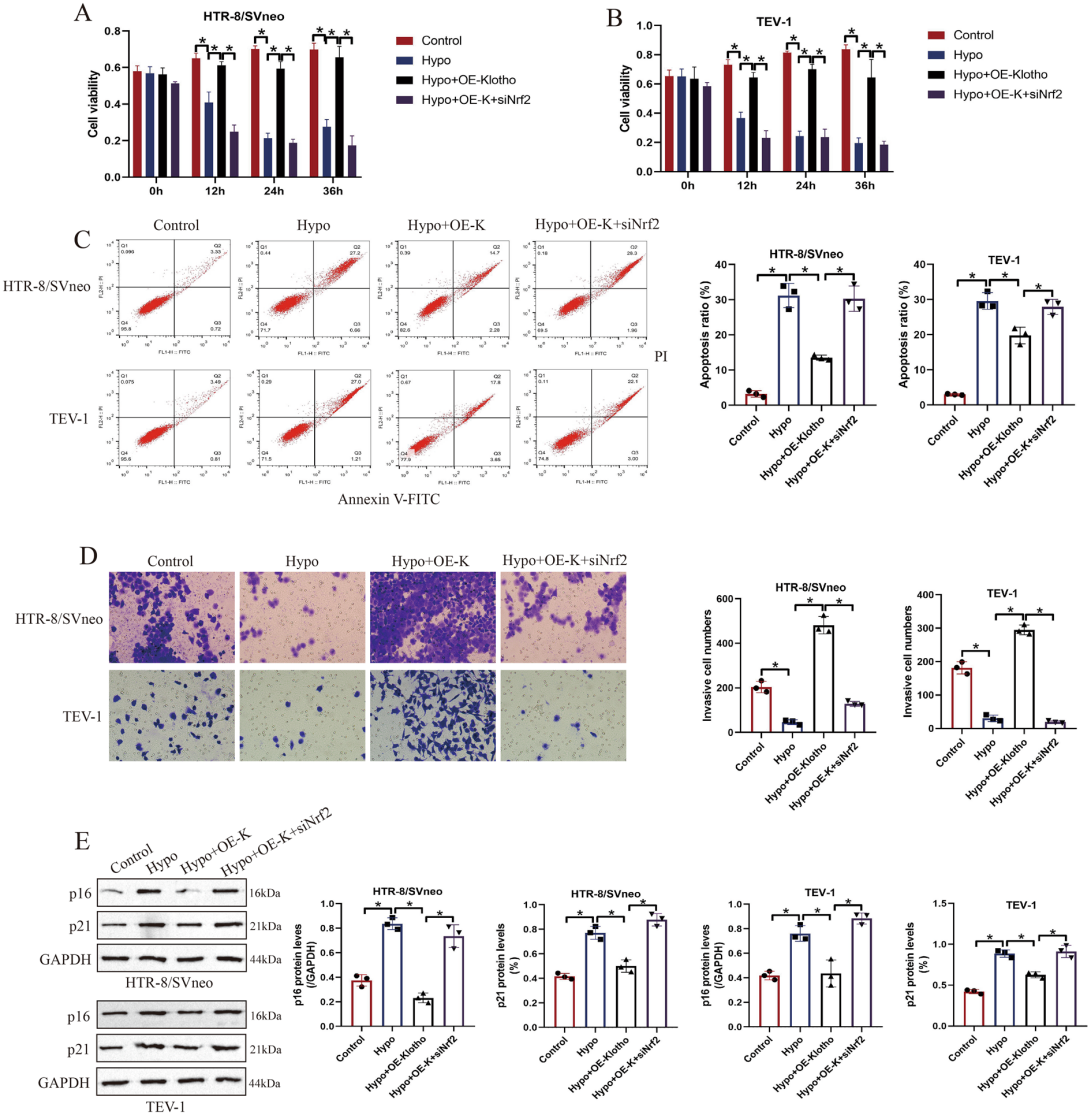
Klotho regulated cell viability, apoptosis, invasion and senescence in hypoxic human trophoblasts by regulating the Nrf2/ARE signal pathway. **A**, **B** The cell viability of human trophoblasts was determined by using the MTT assay. **C** Cell apoptosis in human trophoblasts was determined by using the apoptosis detection kit. **D** Transwell assay was performed to examine cell invasion abilities of human trophoblasts. **E** The cellular senescence-associated proteins (p16 and p21) were examined by performing Western Blot analysis. Each experiment was repeated at least for three times, and *P < 0.05 was considered as statistical significance. Each experiment was repeated at least for three times

Given that Klotho is involved in the regulation of biological functions of hypoxic human trophoblasts, and the anti-oxidant Nrf2/ARE signal pathway is pivotal for the development of PE [8, 35], which can be activated by Klotho overexpression. We next investigated whether Klotho ameliorated hypoxia-induced detrimental effects in human trophoblasts by modulating the Nrf2/ARE signal pathway. To achieve this, the small interfering RNA (siRNA) for Nrf2 (si-Nrf2) was transfected into the HTR-8/SVneo and TEV-1 cells with Klotho overexpression (Fig. 4A, B), which were further cultured in the hypoxic conditions. The results showed that silencing of Nrf2 increased MDA levels (Fig. 4C), whereas suppressed GSH/GSSG ratio (Fig. 4D), and downregulated Nrf2, SOD2 and NQO1 (Fig. 4A, B) to cause excessive oxidative damages in the hypoxic human trophoblasts with Klotho overexpression. The following MTT assay results showed that overexpression of Klotho recovered cell viability in hypoxic human trophoblasts, which were abolished by silencing Nrf2 (Fig. 5A, B). In addition, the FCM assay results supported that Klotho overexpression-induced protective effects in hypoxia-induced human trophoblasts apoptosis were also abrogated by knocking down Nrf2 (Fig. 5C). Then, through performing Transwell assay, we validated that Klotho promoted cell invasion in hypoxic human trophoblasts through upregulating Nrf2 (Fig. 5D). Interestingly, further experiments verified that Klotho Nrf2-dependently downregulated p16 and p21 to suppress cellular senescence in hypoxic human trophoblasts (Fig. 5E). Our data suggested that Klotho Nrf2/ARE pathway-dependently regulated cell apoptosis, mobility and senescence in hypoxia-treated human trophoblasts.

### Klotho overexpression vectors re-activated the anti-oxidant Nrf2/ARE pathway in PE rat models in vivo

**Fig. 6:**
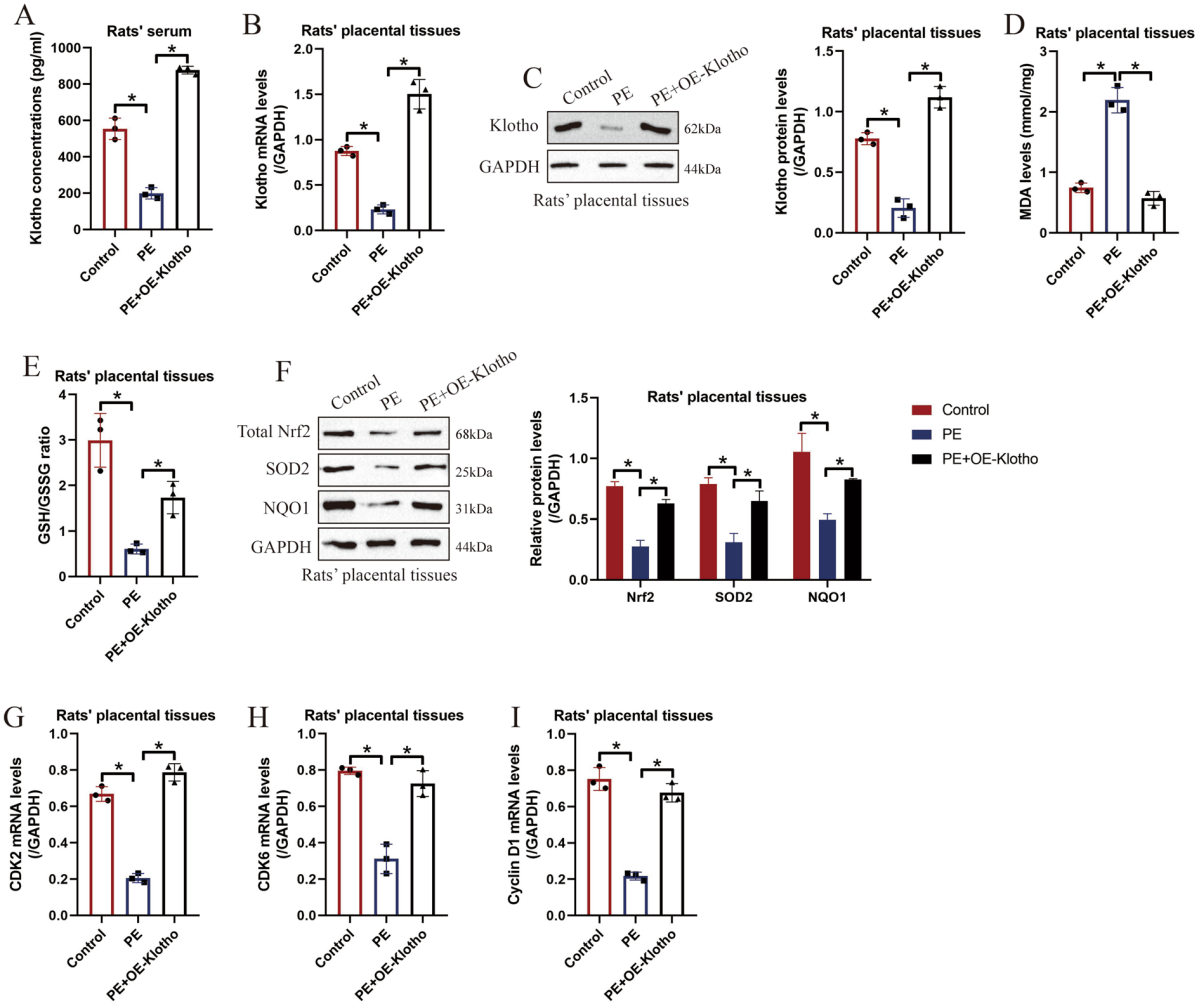
Klotho regulated oxidative damages and cell mitosis in PE rat models in vivo. **A** ELSIA was used to examine Klotho concentrations in PE rats’ serum. The mRNA and protein levels of Klotho in PE rats’ placental tissues were respectively determined by performing **B** Real-Time qPCR and **C** Western Blot analysis. **D** The MDA levels and **E** GSH/GSSG ratio in PE rats’ placental tissues were determined by their commercial kits. **F** The expression levels of total Nrf2, SOD2 and NQO1 in PE rats’ placental tissues were determined by conducting Western Blot analysis. Real-Time qPCR was employed to examined the mRNA levels of **G** CDK2, **H** CDK6 and **I** Cyclin D1 in rats’ placental tissues. Each experiment was repeated at least for three times, and *P < 0.05 was considered as statistical significance. Each experiment was repeated at least for three times

Finally, the reduced uterine perfusion pressure (RUPP) surgery method was employed to establish the PE rat models according to the existed literature [40], and our in vitro results were verified by the in vivo experiments. Specifically, the umbilical cord serum was collected from the normal and PE rats, and the ELISA results showed that Klotho was aberrantly downregulated in the PE rats’ serum compared to the normal counterparts (Fig. 6A). Also, the following experiments supported that the mRNA and protein levels of Klotho were significantly decreased in the PE rats’ placental tissues, as it was respectively determined by Real-Time qPCR (Fig. 6B) and Western Blot analysis (Fig. 6C). Moreover, the PE rats were intraperitoneally administered with Klotho overexpression vectors, and its delivery efficiency in rats’ placental tissues were determined (Fig. 6B, C). The following experiments confirmed that RUPP increased MDA levels (Fig. 6D), whereas suppressed GSH/GSSG ratio (Fig. 6E) and the expression levels of Nrf2, SOD2 and NQO1 (Fig. 6F) to trigger oxidative damages in rats’ placental tissues, which were reversed by overexpressing Klotho (Fig. 6D–F). Moreover, we confirmed that hypoxia decreased the expression levels of CDK2 (Fig. 6G), CDK6 (Fig. 6H) and Cyclin D1 (Fig. 6I) to block cell mitosis in rats’ placental tissues, which were also recovered by upregulating Klotho (Fig. 6G–I). Those data hinted that Klotho suppressed hypoxia-induced oxidative damages and cell cycle arrest to block the development of PE in vivo.

## Discussion

To our knowledge, the pathogenesis of PE is very complicated [1, 2], and dysfunctions of human trophoblasts are the important pathogenetic factor that contribute to the development of PE [3–5]. As previously described, excessive oxidative stress caused malfunctions in trophoblasts aggravates PE development [7, 8], which were verified in our study that MDA levels were elevated, whereas GSH/GSSG ratio and the expression levels of the anti-oxidant genes (Nrf2, SOD2 and NQO1) were downregulated in both PE patients’ placental tissues, hypoxic human trophoblasts and PE rats’ placental tissues, in contrast with their normal counterparts, suggesting that oxidative stress occurred during PE pathogenesis. In recent studies, researchers agree that oxidative stress can affect various biological functions of cells, including cell apoptosis [9, 10], senescence [11, 12] and migration [41, 42], which are closely associated with the development of PE.

. In our study, we verified that hypoxia induced apoptotic cell death, suppressed cell invasion, and upregulated p16 and p21 to exacerbate cellular senescence in human trophoblasts. Also, our in vivo experiments confirmed that downregulated CDK2, CDK6 and Cyclin D1-induced cell cycle arrest happened in PE rats’ placental tissues. Those data suggested that oxidative stress-associated cell apoptosis and senescence in human trophoblasts are involved in the pathogenesis of PE [13]. Previous studies suggest that a large cohort of genes participate in the regulation of PE [16–18], and as one of the important PE-associated genes, the anti-aging gene Klotho is deemed as a sensitive indicator for PE diagnosis and treatment [19, 20]. However, most of the published work only investigate the correlations of Klotho with PE, the detailed functions and underlying mechanisms by which Klotho affects PE pathogenesis are still largely elusive. In our study, we verified that Klotho is aberrantly downregulated in PE patients’ tissues, hypoxia-treated trophoblasts and PE rats’ tissues compared to the normal tissues and cells, suggested that Klotho levels was related with PE progression. Next, our functional experiments revealed that overexpression of Klotho was effective to improve cell viability and invasion, but suppressed oxidative damages, apoptotic cell death and cell senescence in hypoxic human trophoblasts, which were supported by the in vivo experiments that Klotho overexpression recover anti-oxidant effects and cell mitosis in PE rats’ placental tissues. Those data suggested that Klotho was a pivotal gene that suppressed the development of PE.

Based on the existed information, the Nrf2/ARE signal pathway plays critical role in sustaining the homeostasis of oxidative stress [29–31], and this signal pathway has been reported to be involved in regulating PE development [8, 35], which were verified by our results that Nrf2, SOD2 and NQO1 were significantly downregulated in the PE models in vitro and in vivo. Interestingly, previous data hint that the Nrf2/ARE signal pathway can be regulated by Klotho [25, 39], and our data supported this notion that overexpression of Klotho increased the levels of total Nrf2, SOD2 and NQO1, and promoted Nrf2 cytoplasm-to-nucleus translocation to activate the antioxidant Nrf2/ARE signal pathway in hypoxic trophoblasts, which were verified by the animal results that Klotho also re-activated this signal pathway in PE rats’ placental tissues, suggesting that Klotho was capable of activating the Nrf2/ARE signal pathway in human trophoblasts during PE pathogenesis. Moreover, we verified that Klotho regulated PE development via the Nrf2/ARE signal pathway, and our data showed that the regulating effects of Klotho overexpression on oxidative damages, cell apoptosis, senescence and invasion in hypoxic trophoblasts were all abrogated by silencing Nrf2.

## Conclusions

In conclusion, this study firstly investigated the underlying mechanisms by which Klotho involved in the regulation of PE development. Specifically, overexpression of Klotho upregulated Nrf2 and facilitated its translocation from cytoplasm to nucleus, resulting in the activation of the anti-oxidant Nrf2/ARE signal pathway to eliminate oxidative stress, which further suppressed cell apoptosis and senescence, and promoted invasion of human trophoblasts to ameliorate PE development. Our study provided evidences to support that Klotho could be used as ideal biomarkers for the potential treatment of PE in clinic.

## Materials and methods

### Clinical specimens

The human placental tissues and umbilical cord serum were collected from normal volunteers (N = 6), PE patients (N = 5) and PE-SGA patients (N = 8) in The Fifth Affiliated Hospital of Xinjiang Medical University from 2017 to 2021. Clinical data suggested that there existed no differences among the three groups regarding to women age, gestational age and body mass index (BMI), and the clinical characteristics of the enrolled participants were shown in Table 1. All the specimens were collected and immediately placed into the −20 ℃ conditions for further analysis. The informed consent forms had been signed by all the participants, and the clinical experiments were approved by the Ethics Committee Affiliated to The Fifth Affiliated Hospital of Xinjiang Medical University.

**Table 1:** The clinical characteristics of the patients

Features	Normal	PE + PE-SGA	t-values	p-values
Subjects	6	13	–	–
Age	38.23 ± 4.32	36.72 ± 5.11	0.173	0.432
BMI (kg/m^2^)	22.92 ± 3.11	22.47 ± 2.18	0.734	0.332

### Cell culture and treatment

The human trophoblasts (HTR-8/SVneo and TEV-1) were purchased from American Type Culture Collection (ATCC, MA, USA) and cultured in the RPMI-1640 culture medium (Gibco, USA) containing 10% fetal bovine serum (FBS, Gibco, USA) and the antibiotics inducing penicillin (100 U/ml) and streptomycin (100 μg/ml). The cells were placed in the incubator with 5% CO_2_ at 37 ℃ conditions for regulator maintenance. To establish the PE cellular models, the human trophoblasts were cultured in a hypoxic workstation (Electrotek, UK) with 1% O_2_, 5% CO_2_ and 94% N_2_ at 37 ℃ conditions.

### Vectors transfection

The Klotho overexpression vectors and Nrf2 silencing vectors were designed and constructed by Sangon Biotech (Shanghai, China), and the above vectors were delivered into the human trophoblasts by using the Lipofectamine 2000 Transfection reagent (Invitrogen, USA) in keeping with the manufacturer’s instructions. At 24 h post-transfection, the vectors transfection efficiency was determined by Real-Time qPCR and Western Blot analysis, and the vectors-stably expressed trophoblasts were used for further analysis.

### Real-time qPCR analysis

The TRIzol reagent (Signa-Aldrich, USA) was used to extract total RNAs from cells and tissues, and 500 ng of the RNAs were reversely transcribed into complementary DNA (cDNA). The primer sequences for Klotho (F: 5ʹ-GTG CGT CCA TCT GGG ATA CG-3ʹ, R: 5ʹ-TGT CGC GGA AGA CGT TGT T-3ʹ), CDK2 (F: 5ʹ-ATG GAT GCC TCT GCT CTC ACT G-3ʹ, R: 5ʹ-CCC GAT GAG AAT GGC AGA AAG C-3ʹ), CDK6 (F: 5ʹ-CAC CCC AAC GTG GTC AGG TT-3ʹ, R: 5ʹ-TCG GAC CTC ACG GGT GAC TT-3ʹ), Cyclin D1 (F: 5ʹ-TCT ACA CCG ACA ACT CCA TCC G-3ʹ, R: 5ʹ-TCT GGC ATT TTG GAG AGG AAG TG-3ʹ) and GAPDH (F: 5ʹ-AGA AGG CTG GGG CTC ATT TG-3ʹ, R: 5ʹ-AGG GGC CAT CCA CAG TCT TC-3ʹ) were synthesized, and the expression levels of those genes were examined by employing QuantiNova SYBR Green PCR kit (Qiagen, Shanghai, China) in keeping with the manufacturer’s protocols.

### Western blot analysis

The tissues and cells were lysed by using the cold radioimmunoprecipitation (RIPA) buffer (Bio-Rad, CA, USA) on ice, and the cell lysates were heated for 5 min at 95 ℃ for protein denaturation, which were further subjected to 10% SDS-PAGE to separate the proteins according to their molecular weight. The target proteins were then transferred from the gels to PVDF membranes (Millipore, USA), and the proteins-loaded PVDF membranes were blocked with 5% non-fat milk for 40 min at room temperature. Then membranes were subsequently incubated with the primary antibodies against Klotho (1:1500, #ab181373, Abcam, UK), GAPDH (1:2000, #ab8245, Abcam, UK), p16 (1:1500, #ab51243, Abcam, UK), p21 (1:1500, #ab109520, Abcam, UK), Nrf2 (1:2000, #ab62352, Abcam, UK), SOD2 (1:1500, #ab68155, Abcam, UK) and NQO1 (1:2000, #ab80588, Abcam, UK) at 4℃ overnight. The antibodies were washed off and incubated with the horseradish peroxidase-labeled secondary antibody (Cell Signaling Technology, USA) for 1 h at 37 ℃. The ECL system (ThermoFisher Scientific, USA) was used to visualize the protein bands, which were further analyzed by using the Image J software.

### ELISA analysis

The umbilical cord serum was collected from both PE patients in clinic and PE rats in *vivo*, and the concentrations of Klotho were analyzed by using commercial ELISA kit purchased from Abcam (#ab271241, UK) in accordance with the producer’s protocol.

### Detection of cell viability

The MTT assay was performed to examine cell viability by using the commercial kit (Invitrogen, USA) in accordance with the manufacturer’s protocol. Briefly, the human trophoblasts were cultured in the 96-well plates at the concentrations of 1,000 cells per well, and 20 μl of MTT reaction solution was added to the cells for 4 h at 37 ℃. Then, the cell supernatants were removed, and formazan was diluted by 150 μl of DMSO, and the plates were fully vortexed and a microplate reader (ThermoFisher Scientific, USA) was used to detect the optical density (OD) values in each well, which represented the relative cell viability of human trophoblasts.

### Examination of oxidative stress

The malondialdehyde (MDA) levels and GSH/GSSG ratio in tissues and cells were detected by using the corresponding MDA Detection kit (Qiagen, Shanghai, China) and GSH/GSSG Detection reagent (Qiagen, Shanghai, China) following the protocols provided by the manufacturer.

### Apoptosis detection assay

The commercial Annexin V-FITC/PI Apoptosis Detection kit (Abnova, Shanghai, China) was purchased to detect cell apoptosis of trophoblasts. Briefly, the trophoblasts were harvested and suspended in the dilution buffer at the concentration of 1 × 10^6^ cells/ml. Then, 100 μl of cell dilution was mixed with 5 μl Annexin V-FITC and 5 μl PI, and the incubation duration lasted for 15 min at room temperature without light exposure. A FACS Calibur Flow Cytometer (Beckman Coulter, CytoFLEX S, USA) was used to count the Annexin V-FITC/PI-positive apoptotic cell ratio.

### Cell invasion detection assay

The Transwell assay was performed to evaluate cell invasion abilities. In brief, the human trophoblasts were cultured in the upper chamber of the Transwell plates in the FBS-free RPMI-1640 medium, and the lower chamber of the Transwell system was full of RPMI-1640 medium containing 10% FBS. At 24 h post-culture, the middle inserts between the upper and lower chamber were removed and washed by phosphate-buffered saline (PBS) buffer, and fixed with cold methanol for 10 min at room temperature and stained with hematoxylin. The invasive cells were counted under a light microscope (ThermoFisher Scientific, USA) to evaluate the invasive abilities of the trophoblasts.

### Establishment of PE rat models

The PE rat models were established by using the reduced uterine perfusion pressure (RUPP) method according to the protocols recorded in the previous literature [40]. In brief, the rats were purchased from Research Animal Center of The Fifth Affiliated Hospital of Xinjiang Medical University, and the pregnant rats at gestational day 14 were anesthetized, and a middle abdominal incision was created and we further isolated the lower abdominal aorta. A silver surgical clip with 0.203 mm was placed on the lower abdominal aorta above the iliac bifurcation, and both left and right ovarian arteries were clipped by two other silver surgical clips with 0.1 mm. All the animal experiments were approved by the Ethics Committee Affiliated to The Fifth Affiliated Hospital of Xinjiang Medical University.

### Statistical analysis

All clinical, cellular and animal experiments were conducted for at least three times, analyzed by SPSS18.0 software, and visualized by GraphPad Prism 8.0 software. Data was presented as Means ± Standard Deviation (SD) forms, and statistical significance from two groups were analyzed by performing unpaired two-tailed Student’s t-test, and means from multiple groups (above two) were compared by employing one-way ANOVA analysis followed by Tukey’s post hoc test. *P < 0.05 was considered as statistical significance.

## Data Availability

All the data had been included in the paper, and the original raw data could be obtained from the corresponding author with reasonable request.
